# ONE MINUTE TO RECOGNITION: THE AGITATION IN ALZHEIMER’S SCREENER FOR CAREGIVERS (AASC™)

**DOI:** 10.1093/geroni/igad104.3508

**Published:** 2023-12-21

**Authors:** Carolyn Clevenger, Malaak Brubaker, Mehul Patel, Jeffrey Cummings, W Clay Jackson, Jared Stroud, Sue Pechin, George Grossberg

**Affiliations:** Emory University, Atlanta, Georgia, United States; Otsuka Pharmaceutical, Princeton, New Jersey, United States; Otsuka Pharmaceutical, Princeton, New Jersey, United States; School of Integrated Health Sciences, University of Nevada Las Vegas, Las Vegas, Nevada, United States; University of Tennessee College of Medicine, Germantown, Tennessee, United States; OhioHealth, Columbus, Ohio, United States; Alliance for Aging Research, Washington, District of Columbia, United States; Saint Louis University, St. Louis, Missouri, United States

## Abstract

Agitation is a common neuropsychiatric symptom of Alzheimer’s dementia (AD) and both a complex and burdensome aspect of patient care. Caregivers are often first to encounter agitation-associated behaviors and play a pivotal role in communicating these symptoms to healthcare providers (HCPs). However, most caregivers do not identify the breadth of signs/symptoms of agitation and are hesitant to discuss these behaviors with HCPs. There is, therefore, an unmet need for an easy, pragmatic screener to facilitate caregiver recognition of agitation associated with AD (AAD) and HCP discussions. Here, we report on the development of the Agitation in Alzheimer’s Screener for Caregivers (AASC™), a brief screening tool based on the International Psychogeriatric Association (IPA) consensus definition for agitation in cognitive disorders, designed to assist caregivers in recognizing agitation-associated behaviors in patients with AD. Initial draft development involved identification of specific behaviors that were discriminative for AD with/without agitation and representative of the full range of IPA-defined agitation behaviors (i.e., excessive motor activity, verbal aggression, physical aggression) based on current literature and input from 6 opinion leaders. An initial draft was then refined across 2 rounds of cognitive debriefing with caregivers (n=12; mean age=62 years) who described the screening tool as comprehensive yet easy to complete, and perceived it to be valuable, relevant, and easily understood. The AASC™ is a brief, easy-to-use tool supporting the identification and assessment of impact of agitation-associated behaviors. The screener can be completed by a caregiver in < 1 minute at home or in an HCP’s office setting.

